# Expression of astrocyte elevated gene-1 (AEG-1) as a biomarker for aggressive pancreatic ductal adenocarcinoma

**DOI:** 10.1186/1471-2407-14-479

**Published:** 2014-07-03

**Authors:** Yan Huang, Guo-Ping Ren, Chao Xu, Shui-Feng Dong, Ying Wang, Yun Gan, Li Zhu, Tian-Yuan Feng

**Affiliations:** 1 The First People’s Hospital of Yuhang District, 311100 Hangzhou, Zhejiang, China; 2 The First Affiliated Hospital, Zhejiang University School of Medicine, 311100 Hangzhou, Zhejiang, China; 3 The Department of Pathology, The First People’s Hospital of Yuhang District, 311100 Hangzhou, Zhejiang, China

**Keywords:** AEG-1, Biomarker, Prognosis, Pancreatic ductal adenocarcinoma

## Abstract

**Background:**

Altered expression of astrocyte elevated gene-1 (AEG-1) is associated with tumorigenesis and progression. The present study aimed to investigate the clinical and prognostic significance of AEG-1 expression in pancreatic ductal adenocarcinoma (PDAC).

**Methods:**

Quantitative reverse transcriptase polymerase chain reaction (qRT-PCR) and Western blot analyses were employed to assess AEG-1 expression in three pancreatic cancer cell lines and normal pancreatic duct epithelial cells. qRT-PCR and immunohistochemical analyses were performed to detect AEG-1 expression in ten pairs of PDAC and normal pancreas tissues. Immunohistochemistry was then used to examine AEG-1 expression in paraffin-embedded tissues obtained from 105 patients, and its association with clinicopathological parameters including cancer classification was examined. Kaplan-Meier analysis was performed to study the survival rates of patients.

**Results:**

Expression of AEG-1 mRNA and protein was markedly higher in pancreatic cancer cell lines than that in the normal pancreatic duct epithelial cells. AEG-1 expression was evidently upregulated in PDAC tissues compared to that of the matched distant normal pancreas tissues. qRT-PCR data revealed that the tumor/non-tumor ratio of AEG-1 expression was >1.5-fold (up to 6.5-fold). Immunohistochemical data showed that AEG-1 protein was detected in 98.09% (103/105) of PDAC tissues; and they were found to be associated with tumor size (*P* = 0.025), advanced clinical stage (*P* = 0.004), T classification (*P* = 0.006), N classification (*P* = 0.003), and M classification (*P* = 0.007). Furthermore, Kaplan-Meier analysis showed that patients with high AEG-1-expressed PDAC had shorter overall survival. A multivariate Cox regression analysis revealed that clinical stage, T classification, and AEG-1 expression were the independent prognostic predictors for PDAC.

**Conclusions:**

This study suggests that AEG-1 protein was highly expressed in PDAC and associated with poor prognosis of the patients.

## Background

Pancreatic cancer is one of the most aggressive gastrointestinal malignancies, accounting for the fourth most common cause of cancer-related deaths in the United States and the eighth in the world [1]. Pancreatic ductal adenocarcinoma (PDAC) is the most common type of pancreatic cancer and is frequently diagnosed at locally advanced or metastatic disease, leading to an extremely poor prognosis clinically [2]. To date, surgery is the only curable treatment for PDAC, as it usually is resistant to conventional chemotherapy and radiation therapy [3]. Although recent molecular analyses of precursor lesions revealed an association between gene alterations and carcinogenesis of PDAC, the molecular mechanisms that regulate the aggr7essive behavior of PDAC still remain to be clarified [4]. The actual etiology of PDAC remains unclear, and a number of risk factors are associated with PDAC development including family history; chronic pancreatitis; diabetes; obesity; and consumption of alcohol, tobacco, sugar-sweetened drinks, and red meat [5]. PDAC development, like all other cancers, involves multiple genetic alterations such as oncogene activation and tumor-suppressor gene dysfunction [2]. Thus, it is of great value to better understand the etiology, identify valuable diagnostic and prognostic markers, and explore novel therapeutic strategies for this deadly disease.

Astrocyte elevated gene-1 (AEG-1) was discovered as a novel protein induced by human immunodeficiency virus-1 or tumor necrosis factor-α in primary human fetal astrocytes [6-8]. AEG-1 is an oncogene and is aberrantly elevated in different human cancers such as breast cancer, glioblastoma cell migration, esophageal squamous cell carcinoma, prostate cancer, and hepatocellular carcinoma [9-15]. As a downstream target of Ha-Ras, AEG-1 plays an essential role in promoting tumorigenesis, invasion, metastasis, and angiogenesis [16]. Molecularly, AEG-1 promotes tumor cell proliferation by suppressing forkhead box protein O1, induces serum-independent cell growth, suppresses apoptosis through activation of PI3K-Akt signaling [16-20], and increases anchorage-independent growth of non-tumorigenic astrocytes through activation of PI3K-Akt and nuclear factor-kappa B (NF-κB) pathway [17,21]. Overexpression of AEG-1 promotes tumorigenesis and progression by activating ERK, Akt and p38 MAPK pathways by phosphorylation in hepatocellular carcinoma [9]. However, knockdown of AEG-1 expression could inhibit prostate cancer progression [14]. AEG-1 can regulate human malignant glioma invasion through upregulation of matrix metalloproteinase-9 and activation of NF-κB signaling pathway [11,18,21,22]. These findings suggest that AEG-1 plays a dominant role in the development and progression of diverse cancers. In this study, the expression of AEG-1 messenger ribonucleic acid (mRNA) and protein in PDAC tissues were examined for association with clinicopathological and prognostic significance.

## Methods

### Cell lines and culture

Pancreatic cancer cell lines were obtained from American Type Culture Collection (Manassas, VA). AsPC-1 was originally isolated from ascites of a patient with a Grade 2 PDAC, while Mia Paca-2 and Panc-1 were from patients with poorly-differentiated (G3) primary PDAC, Capan-1 was isolated from a lymph node metastasis of a PDAC patients, BxPC-3 was isolated from a patient with pancreas ductal carcinoma in situ. In contrast, HPDE6 was isolated from normal epithelial tissue of pancreatic duct. AsPC-1 cells were maintained in RPMI-1640 medium containing 10% fetal bovine serum (FBS), penicillin (50 U/ml), and streptomycin (50 U/ml). MiaPaca-2 cells were maintained in Dulbecco’s modified Eagle’s medium (DMEM) containing 10% FBS, 2.5% horse serum (HS), penicillin (50 U/ml), and streptomycin (50 U/ml). Panc-1 and BxPC-3 cells were maintained in DMEM containing 10% FBS, penicillin (50 U/ml), and streptomycin (50 U/ml). Capan-1 cells were maintained in a Dulbecco’s Modified Eagle’s Medium supplemented with 20% fetal calf serum, penicillin (50 U/ml) and streptomycin (50 U/ml). HPDE-6 cells were routinely cultured in keratinocyte serum-free (KSF) medium supplemented by epidermal growth factor and bovine pituitary extract. All cell culture supplements, FBS, and HS were obtained from Gibco BRL (Grand Island, NY).

### Tissue specimens

Fresh PDAC tissue specimens obtained from 10 patients and the corresponding normal tissues were obtained from the First People’s Hospital of Yuhang District and the First Affiliated Hospital, Zhejiang University School of Medicine between January 2011 and December 2012. Additionally, formalin-fixed and paraffin-embedded PDAC tissue samples were also obtained from 105 patients between January 2010 and December 2012. All tissue specimens were taken from patients who underwent pancreatic cancer surgery, and the patients did not receive any preoperative tumor therapy. Normal pancreas tissue adjacent to carcinoma required at least 5 cm away from the tumor edge. Clinical and pathological classification and staging were determined per the World Health Organization (WHO) classification criteria [23]. Clinicopathological data of these 105 patients are summarized in Table 1. Ten pairs of fresh PDAC and matched distant non-cancerous pancreatic tissues were frozen and stored in liquid nitrogen until use. This study was approved by the Ethic Committee of the First People’s Hospital of Yuhang District and the Ethic Committee of the First Affiliated Hospital, College of Medicine, Zhejiang University, and each patient signed an informal consent form before enrolled into the study.

**Table 1:** Association of AEG-1 expression with clinicopathological characteristics of PDAC patients

**Characteristics**		**AEG-1 expression**		
		**Low or none N (%)**	**High N (%)**	**Chi-square test, *P*-value**
Gender	Male	31 (29.52)	32 (30.48)	0.172
	Female	15 (14.29)	27 (25.71)	
Age (years)	< 60	23 (21.90)	20 (19.05)	0.096
	≥ 60	23 (21.90)	39 (37.15)	
Localization	Head of the pancreas	28 (26.67)	46 (43.81)	0.057
	Body/tail of the pancreas	18 (17.14)	13 (12.38)	
Tumor size	≤ 2	10 (9.52)	4 (3.81)	0.025
	> 2	36 (34.29)	55 (52.38)	
Clinical Stage	I	19 (18.10)	12 (11.43)	0.004
	II	6 (5.71)	4 (3.81)	
	III	15 (14.29)	25 (23.81)	
	IV	6 (5.71)	18 (17.14)	
T classification	T1	10 (9.52)	4 (3.81)	0.006
	T2	29 (27.62)	33 (31.43)	
	T3	3 (2.85)	11 (10.48)	
	T4	4 (3.81)	11 (10.48)	
N classification	N0	29 (27.62)	20 (19.05)	0.003
	N1	17 (16.19)	39 (37.14)	
M classification	No	43 (40.95)	43 (40.95)	0.007
	Yes	3 (2.86)	16 (15.24)	
Histological Grades	Well-differentiated	1 (0.95)	2 (1.90)	0.052
	Moderately differentiated	40 (30.10)	45 (42.86)	
	Poorly differentiated	4 (3.81)	13 (12.38)	
Histological Types	Classical ductal adenocarcinoma	43 (40.95)	48 (45.73)	0.097
	Adenosquamous carcinomas	2 (1.90)	5 (4.76)	
	Undifferentiated carcinomas	0 (1.00)	4 (3.81)	
	Mixed ducal-neuroendocrine carcinoma	1 (0.95)	2 (1.90)	
Alcohol drinking	No	24 (22.86)	40 (38.09)	0.104
	Yes	22 (20.95)	19 (18.10)	
Tobacco smoking	No	28 (26.67)	28 (26.67)	0.172
	Yes	18 (17.14)	31 (29.52)	

### RNA isolation and quantitative reverse transcriptase polymerase chain reaction (qRT-PCR)

Total cellular RNA was isolated from tissue samples using a Trizol reagent (Invitrogen, Carlsbad, CA) per the manufacturer’s instructions. These RNA samples were then treated with RNase-free DNase, and 2 μg of RNA sample of each patient was subjected to complementary deoxyribonucleic acid (cDNA) synthesis using random hexamers. PCR amplification was performed to detect AEG-1 cDNA using AEG-1–specific primers; and the PCR conditions included the following: initial denaturation of samples at 95°C for 10 min, followed by 40 cycles of denaturation at 95°C for 10 s, primer annealing at 60°C for 60 s, followed primer extension at 72°C for 30 s, final extension at 72°C for 5 min, and storage at 4°C. qPCR was then employed to determine the fold of increase of AEG-1 mRNA in each of the primary PDAC relative to the adjacent pancreatic tissues taken from the same patient. Expression data were normalized to the geometric mean of housekeeping gene glyceraldehyde 3-phosphate dehydrogenase (GAPDH) to control the variability of expression levels. PCR primers were designed using the Primer Express v 2.0 software (Applied Biosystems, Foster city, CA). The primers for AEG-1 were 5′-CGTGATAAGGTGCTGACTGATTC-3′ and 5′-CAGGAAATGATGCGGTTGTAAG-3′. The primers for GAPDH were 5′-GGGAAACTGTGGCGTGAT-3′ and 5′-GAGTGGGTGTCGCTGTTGA-3′. These primers were synthesized by Sangon Ltd (Shanghai, China). Expression data were calculated as 2-[(Ct of AEG-1) – (Ct of GAPDH)], where Ct represents the threshold cycle for each transcript.

### Protein extraction and Western blot

The proteins were transferred to nitrocellulose membranes (Amersham Pharmacia Biosciences, Freiburg, Germany). AEG-1 was detected by using a rabbit polyclonal anti-AEG-1 antibody (Abcam, Heidelberg, Germany) diluted at 1:500, and the enhanced chemiluminescence plus Western blot detection system (Amersham Pharmacia Biosciences). After detection, the blots were stripped, and anti-α-tubulin was detected using a mouse monoclonal antibody (Sigma, Saint Louis, MI) diluted 1:1,000. The secondary antibody was diluted 1:5,000 in both cases.

### Immunohistochemistry

Immunohistochemical analysis was performed to detect AEG-1 protein expression in 105 PDAC tissues. In brief, paraffin-embedded tissue blocks were cut into 4-μm thick sections and baked at 65°C for 30 min. The sections were then deparaffinized and rehydrated for antigenic retrieval by submerging the sections in the ethylenediaminetetraacetic acid buffer and microwaved for 8 min. The sections were then incubated in 3% hydrogen peroxide in methanol to quench the endogenous peroxidase activity, followed by incubation with 1% bovine serum albumin to block nonspecific binding. After that, a rabbit anti-AEG-1 antibody (1:200; Abcam) was added onto the section and incubated overnight at 4°C. For negative controls, the first antibody was replaced with a normal nonimmune serum.

The immunostained tissue sections were then either reviewed and scored blindly by two independent pathologists or subjected to the mean optical density (MOD) quantification. For semi-quantitative analysis, the score of each tissue section was based on both the proportion of positively stained tumor cells and the intensity of staining. The proportion of tumor cells was scored as follows: 0 (no positive tumor cells), 1 (<10% positive tumor cells), 2 (10-50% positive tumor cells), and 3 (>50% positive tumor cells). The intensity of staining was graded per the following criteria: 0 (no staining); 1 (weak staining = light yellow), 2 (moderate staining = yellow brown), and 3 (strong staining = brown). The staining index (SI) was calculated as staining intensity score x proportion of positive tumor cells. Expression of AEG-1 in normal pancreas epithelium and malignant lesions was determined by SI, which was scored as 0, 1, 2, 3, 4, 6, and 9. Cutoff values for AEG-1 were chosen on the basis of a measure of heterogeneity with the log-rank test with respect to overall survival. An optimal cutoff value was identified as follows: SI score of ≥ 4 was used to define tumors with high AEG-1 expression and ≤ 3 was defined as tumors with low expression of AEG-1 protein.

For the MOD quantification, the stained sections were evaluated at 200× magnification using the SAMBA 4000 computerized image analysis system with Immuno 4.0 quantitative program (Image Products International, Chantilly, VA). Ten representative microscopic fields of each tumor sample were analyzed to determine the MOD, which represented the concentration of the stain or proportion of positive pixels within the whole tissue. A negative control for each staining batch was used for background subtraction in the quantitative analysis. The data were then statistically analyzed using Student’s *t*-test to determine the differences in average MOD values between tumor and normal tissues.

### Statistical analyses

All statistical analyses were performed by using the SPSS 13.0 statistical software package (SPSS, Chicago, IL, USA). Comparisons between groups for statistical significance were performed with a two-tailed paired Student’s *t* test. The chi-square test was used to analyze association between AEG-1 expression and clinicopathological data. Bivariate correlations between variables were calculated by Spearman’s correlation coefficients, and Scatter was used to represent the relationship between two variables. Survival curves were plotted using Kaplan-Meier method and compared using log-rank test. Survival data were evaluated using univariate and multivariate Cox regression analyses. *P* < 0.05 was considered statistically significant.

## Results

### Upregulation of AEG-1 expression in PDAC cells and tissues

**Figure 1:**
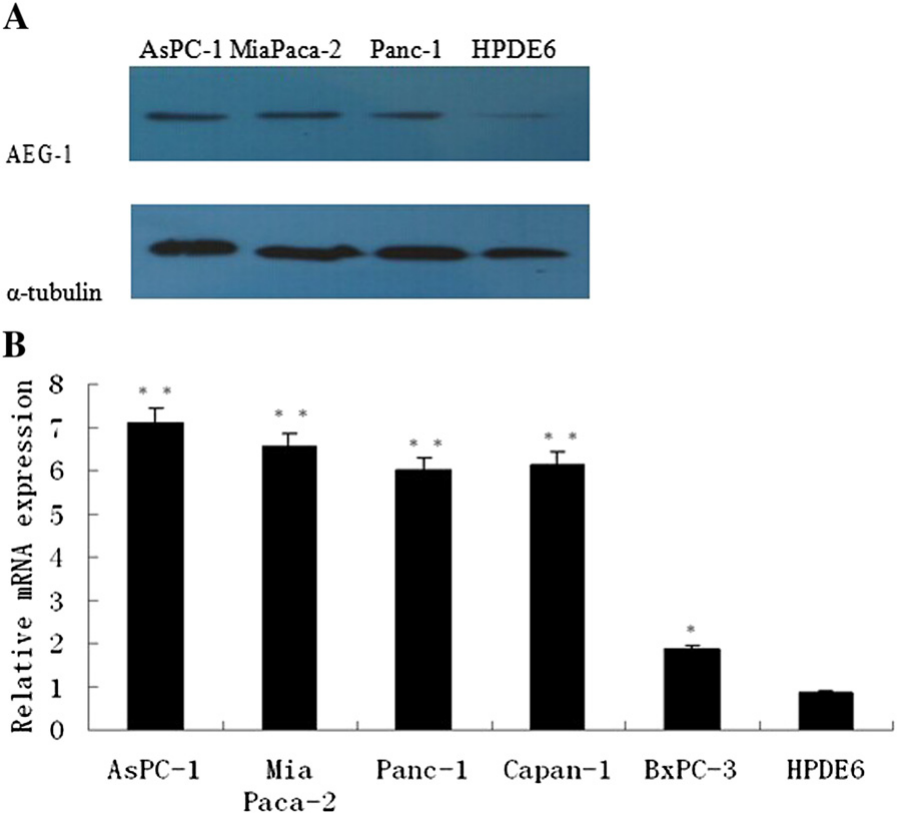
**Analysis of AEG-1 expression in PDAC cell lines. (A)** Western blot. **(B)** qRT-PCR.

**Figure 2:**
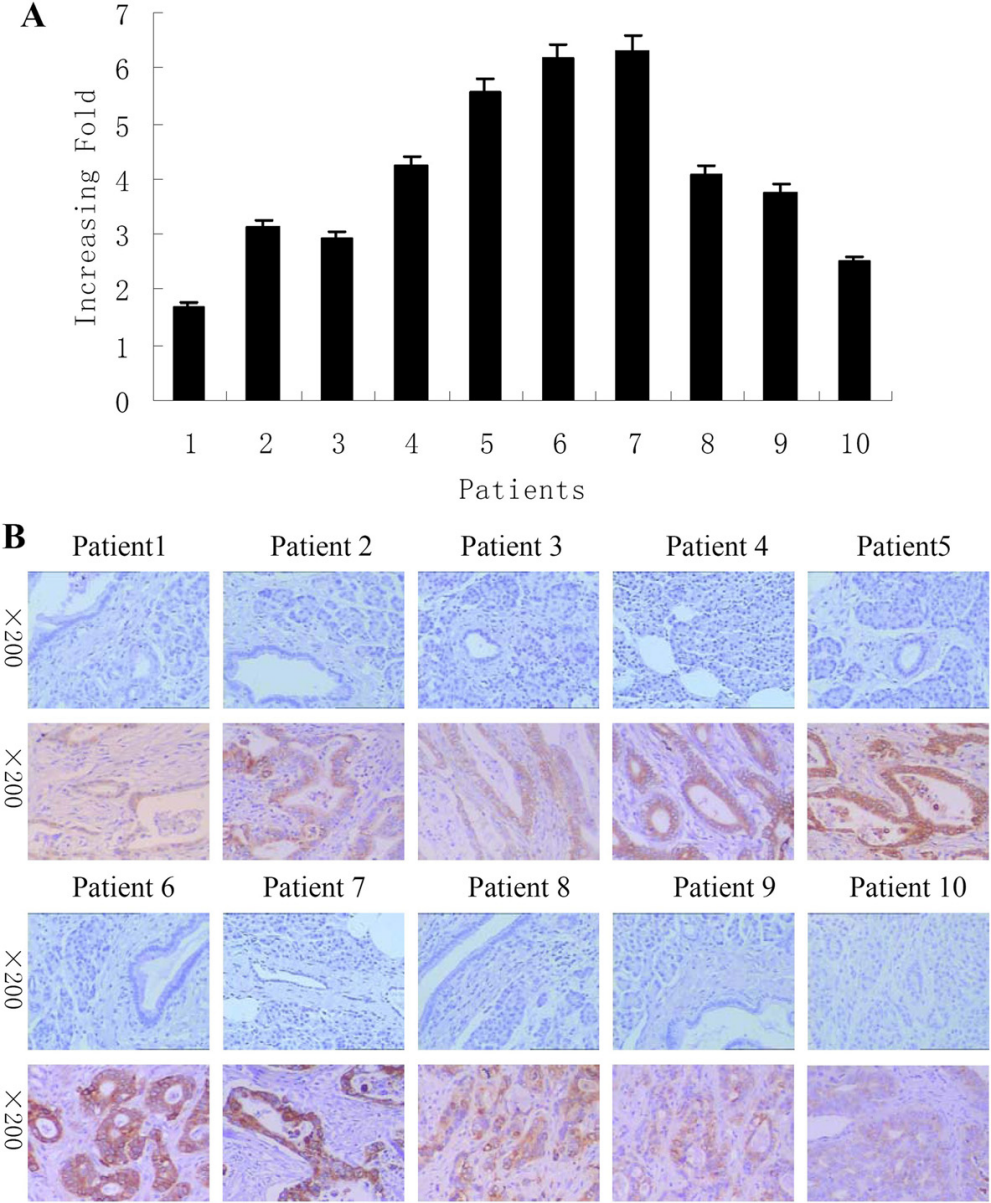
**Upregulation of AEG-1 expression in PDAC tissues. (A)** qRT-PCR analysis of AEG-1 expression in each of the ten PDAC tissues (T) and distant non-cancerous tissues (N). GAPDH was used as an internal control. Columns, mean from three parallel experiments; bars, SD. **(B)** Immunohistochemical analysis of AEG-1 expression in each of the ten PDAC tissues (lower panel) and distant non-cancerous tissues (upper panel).

qRT-PCR data showed that all PDAC lines exhibited significantly higher (up to 8.1-folds) levels of AEG-1 mRNA compared to the normal pancreatic ductal epithelial cells, while Western blot analysis showed that AEG-1 protein was highly expressed in all pancreatic cancer cell lines including AsPC-1, Mia Paca-2, and Panc-1. However, it was weakly expressed in normal pancreatic ductal epithelial cell HPDE6 (Figure 1A and B).After that, this finding was confirmed in 10 cases of paired primary PDAC and adjacent non-cancerous tissues. The data showed that AEG-1 mRNA was significantly upregulated in PDAC tissues of all ten patients, whereas most of the ten normal tissues only had trace amounts of detectable AEG-1 mRNA (Figure 2). The tumor/non-tumor (T/N) ratio of AEG-1 mRNA expression was >1.5-fold in all cases, up to about 6.5-fold induction (Figure 2A). Meanwhile, the expression of AEG-1 protein was also upregulated in all ten PDAC tissue samples compared to that of their matched distant noncancerous tissues by immunohistochemistry (Figure 2B).

### Overexpression of AEG-1 protein in archived PDAC samples

**Figure 3:**
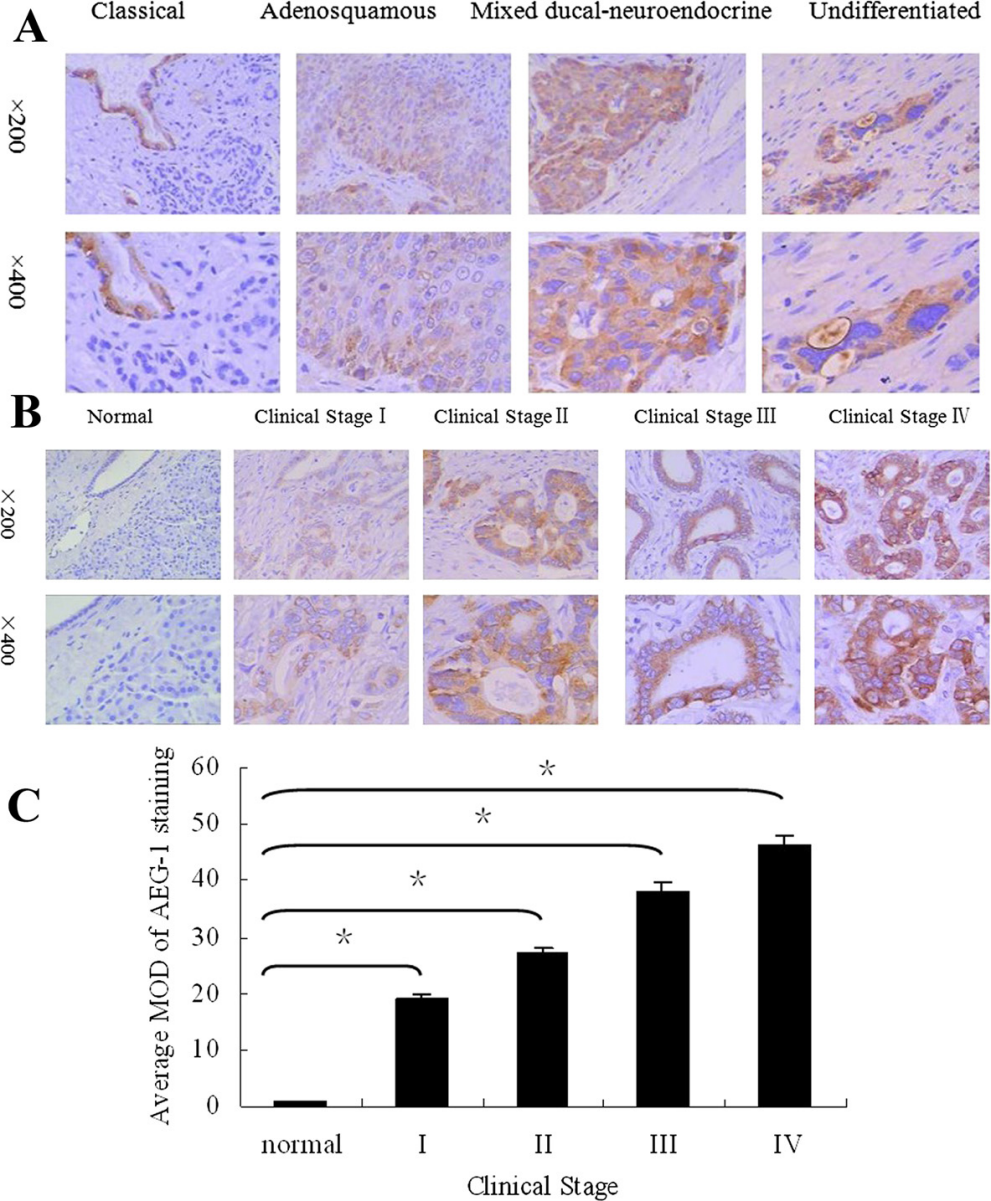
**Immunohistochemical analysis of AEG-1 protein overexpression in archived paraffin-embedded PDAC tissue sections. (A)** Representative images of immunohistochemical analyses of AEG-1 expression in four different histological types of PDAC. **(B)** Representative images of immunohistochemical analyses of AEG-1 expression in normal pancreas and PDAC tissue specimens. **(C)** Statistical analyses of the average MOD of AEG-1 staining between normal pancreas and PDAC tissues specimens of different clinical stages. *P < 0.05.

Immunohistochemistry was performed to determine AEG-1 expression in 105 paraffin-embedded, archived PDAC tissue samples, including four histological types of PDAC: classical ductal adenocarcinoma, adenosquamous carcinomas, undifferentiated carcinomas, and mixed ducal-neuroendocrine carcinoma. AEG-1 expression was detected in 98.09% (103/105) of these PDAC samples, and was found to be mainly localized in the cytoplasm of tumor cells. As shown in Figure 3A, quantitative immunohistochemical data revealed that the MOD valuses of AEG-1 was upregulated in all the examined histological types of PDAC compared to their distant normal tissues.

Figure 3B shows representative immunohistochemically-stained tumor sections of each of the four WHO stages of PDAC. Moderate to strong cytoplasmic staining of AEG-1 protein was observed in tumor cells in these PDAC tissues. But, weak or negative signals were observed in normal tissues (Figure 3B). Quantitative immunohistochemical data revealed that the MOD values of AEG-1 staining in all PDAC tissues were higher than that in normal tissues, and the values increased along with progression of tumor stages I to IV (*P* = 0.004, Figure 3C).

**Figure 4:**
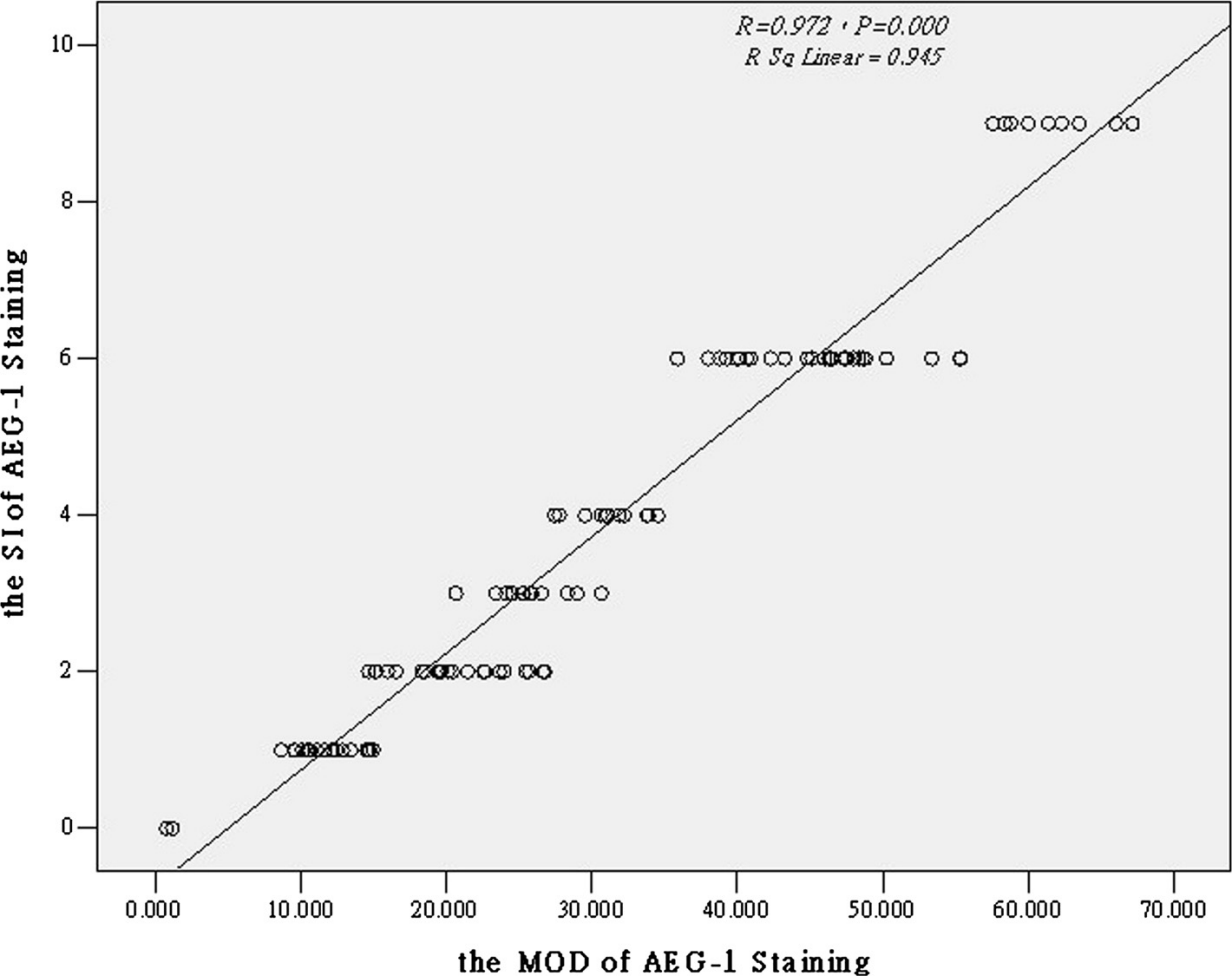
**Scatterplot of the SI staining and the MOD staining of AEG-1.** The SI staining and the MOD staining of AEG-1 expression correlations between variables were calculated by Spearman’s correlation coefficients. ^*^P < 0.05.

To account for the inconsistency in intensity of immunostained sections, we made a scatterplot of the SI staining and the MOD staining of AEG-1.We found that the SI staining and the MOD staining has positive correlation (Figure 4, *R* = 0.972, *R Sq Linear =0.945*, *P* = 0.0001), which showed the SI score is credible; thus, the subsequent statistical analyses used the SI of AEG-1 staining data.

### Increased AEG-1 expression associated with clinicopathological data from patients with PDAC

AEG-1 expression analyzed semi-quantitatively (See the methods section) was strongly associated with clinical stage (*P* = 0.004), T classification (*P* = 0.006), N classification (*P* = 0.003), and distant metastasis (*P* = 0.007; Table 1). Spearman correlation analysis showed that high level of AEG-1 expression was strongly associated with advanced clinical stage (*R* = 0.430, *P* = 0.000), advanced T classification (*R* = 0.284, *P* = 0.002), lymph node involvement (*R* = 0.270, *P* = 0.003), and distant metastasis (*R* = 0.251, *P* = 0.005; Table 2). However, no associations were found between AEG-1 expression and other clinical features such as age, gender, histological variant, history of alcohol consumption, and tobacco smoking.

**Table 2:** Spearman correlation analysis of AEG-1 vs. clinical pathologic factors

**Variables**	**AEG-1 expression level**	
	**Correlation coefficient**	***P*-value**
Clinical staging	0.430	0.000
T classification	0.284	0.002
N classification	0.270	0.003
M classification	0.251	0.005

### AEG-1 expression associated with poor prognosis of patients with PDAC

**Figure 5:**
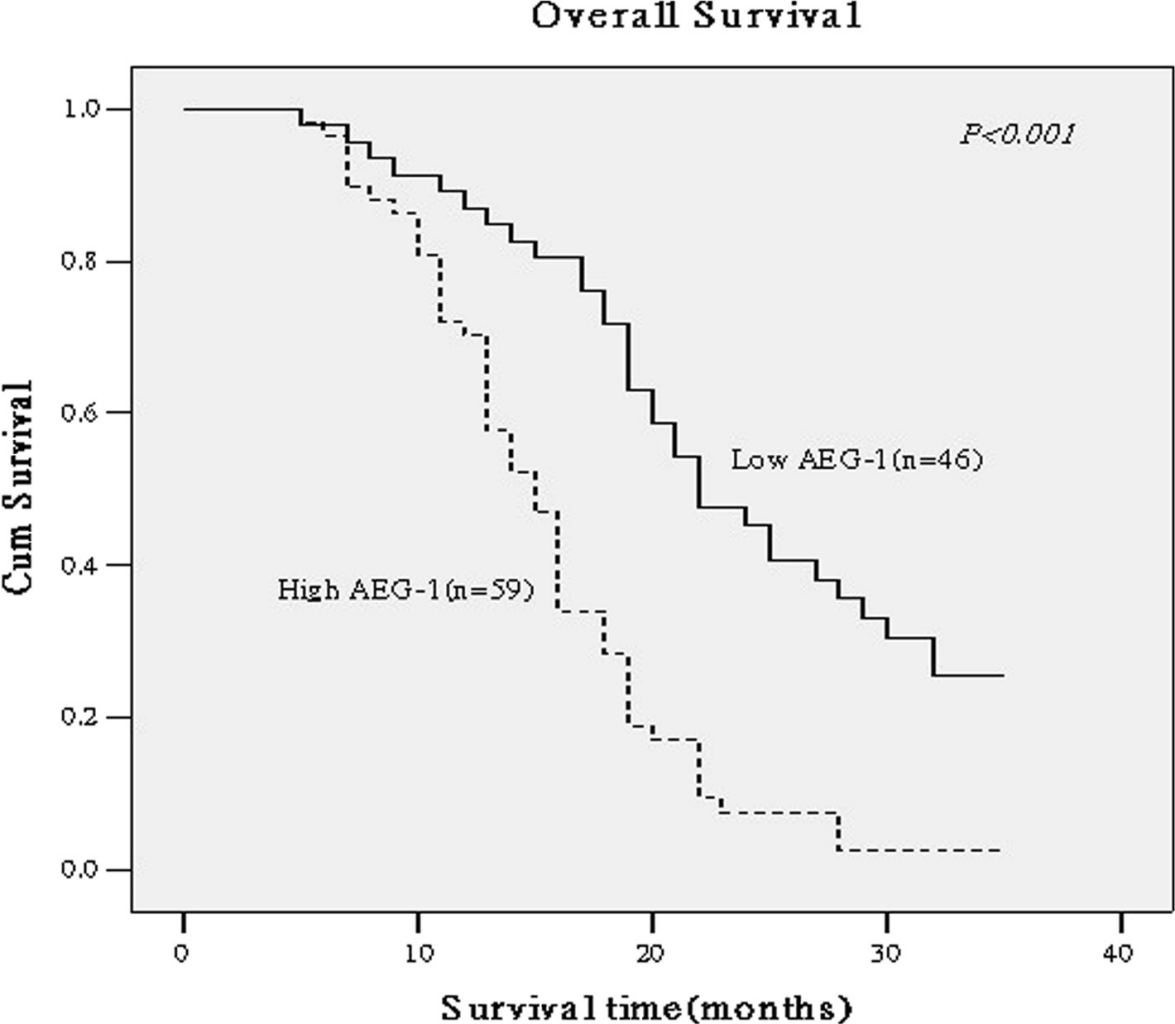
**Kaplan-Meier curves of AEG-1 expression against overall survival of PDAC patients.** The data were analyzed using a log-rank test between patients with low AEG-1 expressed PDAC (full line) versus high AEG-1-expressed PDAC (dotted line). The cumulative 2-year survival rate was 38.09% in patients with low AEG-1-expressed PDAC (n = 46) compared to only 7.84% in patients with high AEG-1-expressed PDAC (n = 59).

Spearman correlation analysis revealed that high levels of AEG-1 expression analyzed semi-quantitatively (See the Methods section) were associated with shorter overall survival of patients with PDAC (*P* < 0.001, correlation coefficient = -0.368). Moreover, Kaplan-Meier analysis showed that patients with low AEG-1-expressed PDAC had longer overall survival compared to those with high AEG-1-expressed PDAC (*P* < 0.001 by a log-rank test; Figure 5). The cumulative 2-year survival rate was 38.09% (95% confidence interval: 0.565–0.913) in patients with low AEG-1-expressed PDAC compared to only 7.84% (95% confidence interval: 0.403–0.697) in high AEG-1-expressed PDAC. In addition, the multivariate Cox regression analysis showed that clinical stage, T classification, and AEG-1 expression were independent prognostic predictors for PDAC (Table 3).

**Table 3:** Univariate and multivariate analyses of various prognostic parameters in patients with PDAC

	**Univariate analysis**			**Multivariate analysis**		
	**No. patients**	***P***	**Regression coefficient (SE)**	***P***	**Relative risk**	**95% confidence interval**
T classification						
T1	14	0.000	1.833 (0.243)	0.003	1.814	1.191-3.262
T2	62					
T3	14					
T4	15					
Clinical staging						
I	31	0.000	2.357 (0.255)	0.001	2.210	1.429-3.887
II	10					
III	40					
IV	24					
Expression of AEG-1						
Low expression	46	0.001	2.588 (0.303)	0.002	2.173	1.288-4.055
High expression	59					

## Discussion

The results obtained in this study showed that expression of AEG-1 mRNA and protein was upregulated in PDAC cell lines and tissues. The results also showed that elevated expression of AEG-1 protein was associated with tumor size, clinical stage, T classification, lymph node, and distant metastases of PDAC. Expression of AEG-1 protein also associated with poor prognosis and reduced survival of patients with PDAC. Moreover, the multivariate Cox regression analysis showed that clinical stage, T classification, and AEG-1 expression were independent prognostic predictors for PDAC. Further studies would verify the results of the present study before AEG-1 could be used as a biomarker for prediction of PDAC prognosis. Such studies would also investigate the role and function of AEG-1 in PDAC.

AEG-1 is an Ha-Ras–regulated gene, which plays an essential role in promotion of tumorigenesis and cancer invasion, metastasis, and angiogenesis [16]. A number of studies have confirmed the potential role of AEG-1 in the development and progression of human cancers [9-15,24-27]. Nonetheless, it remains to be clarified whether AEG-1 expression is in parallel with the course of carcinogenesis and cancer progression or AEG-1 is the driver for tumor development and progression. In either way, AEG-1 could be used as an indicator of cancer progression, but a mechanistic study would define the role of AEG-1 in PDAC.

In the current study, expression of AEG-1 mRNA and protein was upregulated in PDAC cell lines as well as PDAC tissues. After that, AEG-1 expression was detected in PDAC tissue specimens of 105 patients. 103 out of 105 (98.09%) specimens of PDAC tissues had moderate to strong cytoplasmic staining of AEG-1 protein, whereas there was no significant staining of AEG-1 detected in the distant noncancerous pancreatic epithelial cells. This supported the role of AEG-1 in the development and progression of PDAC. Moreover, it is particularly noteworthy per the study results that AEG-1 has been found to be only localized in the cytoplasm of cancer cells. This observation coincides with the most previous reports that overexpression of AEG-1 could result in the localization of the protein in the cytoplasm [28]. However, Emad et al [18,21] found that the cytoplasm and nuclear staining of AEG-1 associated with tumor progression, metastasis and neurodegeneration. In breast cancer, nuclear staining of AEG-1 tends to become more common in lesions from patients with more advanced disease stages [12]. The authors found that occasional nuclear staining of AEG-1 was detected in clinical stage II samples, while stage III sections displayed noticeably increased AEG-1 nuclear localization. A large proportion of caner cells in liver metastases revealed AEG-1 translocation to the nucleus [12]. Emad [21] suggested that AEG-1 might interact with the NF-κB complex and correspond with the nuclear translocation of p65, but suspected that AEG-1 activation of NF-κB was possible by degradation of IκBα. In addition, it was recently reported that the knockdown of AEG-1 expression attenuated the constitutive activity of NF-κB in parallel with depletion in NF-κB-regulated genes [29]. Therefore, the present study data further support the latter possibility. However, further studies are needed to verify the role of AEG-1 at different cellular localizations in the development and signal transduction of PDAC.

Further analysis in the study showed a significant association of AEG-1 expression with advanced clinical staging, and T, N, and M classification. This suggested that AEG-1 might be useful as a biomarker to identify subsets of patients with PDAC who had more aggressive disease. Patients with high AEG-1-expressed PDAC had only a 7.84% cumulative 2-year survival rate, which was significantly lower than that in patients with low AEG-1–expressed PDAC (38.09%). The multivariate Cox regression analysis showed that clinical stage, T classification, and AEG-1 expression were independent prognostic predictors for PDAC.

The *ex vivo* analysis of AEG-1 expression could be a limitation of this study. An *in vitro* mechanistic study of AEG-1 knockout or transgenic animal models in PDAC cell would be important for further understanding of the functional significance of AEG-1 in PDAC development and progression.

## Conclusions

Our current study demonstrated that up-regulation of AEG-1 expression was associated with worse survival of PDAC patients by showing that AEG-1 protein level is an independent prognostic predictor for PDAC patients. Thus, further study will confirm our current data before used in clinical practice.

## Abbreviations

AEG-1: Astrocyte elevated gene-1; cDNA: Complementary deoxyribonucleic acid; DMEM: Dulbecco’s modified Eagle’s medium; FBS: Fetal bovine serum; GAPDH: Housekeeping gene glyceraldehyde 3-phosphate dehydrogenase; HS: Horse serum; KSF: Keratinocyte serum-free medium; MOD: Mean optical density; mRNA: Messenger ribonucleic acid; NF-κB: Nuclear factor-kappa B; PDAC: Pancreatic ductal adenocarcinoma; qRT-PCR: Quantitative reverse transcriptase polymerase chain reaction; SI: Staining index; WHO: World health organization.

## Competing interests

The authors declare that they have no competing interests.

## Authors’ contributions

YH participated in research design, carried out the RNA isolation and qRT-PCR experiments, and drafted the manuscript; GPR collected tissue specimens and patient information. CX and GPR carried out data collection by reading and diagnosing histologic sections. SFD performed cell culture and Western blot. SFD and YW performed immunohistochemistry. YG and LZ performed the statistical analyses. TYF conceived of the study, and participated in research design and coordination of data collection and analyses and helped to draft the manuscript as well. All authors have read and approved the final version of the manuscript.

## Pre-publication history

The pre-publication history for this paper can be accessed here:

http://www.biomedcentral.com/1471-2407/14/479/prepub
